# The origins of a research community in the Majengo observational cohort study, Nairobi, Kenya

**DOI:** 10.1186/1471-2458-10-630

**Published:** 2010-10-21

**Authors:** Sunita VS Bandewar, Joshua Kimani, James V Lavery

**Affiliations:** 1 Program on Ethics and Commercialization, McLaughlin-Rotman Centre for Global Health, University Toronto Network and University of Toronto, 101 College St., Toronto, Ontario M5G 1L7 Canada; 2 Kenya AIDS Control Project, Institute of Tropical and Infectious Diseases, UNITID Building, College of Health Sciences, University of Nairobi, P.O. Box 19676-00202. Nairobi, Kenya; 3 Centre for Research on Inner City Health & Centre for Global Health Research, Keenan Research Centre in the Li Ka Shing Knowledge Institute of St. Michael's Hospital, 30 Bond St. Toronto, Ontario M5B 1W8, Canada; 4 Dalla Lana School of Public Health and Joint Centre for Bioethics, University of Toronto, Toronto, M5B 1W8, Canada

## Abstract

**Background:**

Since the 1980s the Majengo Observational Cohort Study (MOCS) has examined sexually transmitted infections, in particular HIV/AIDS, in a cohort of sex workers in Majengo, an impoverished urban village in Nairobi, Kenya. The MOCS investigators have faced criticism since the women have remained in the sex trade for the duration of their participation in the study, prompting concerns about exploitation. Yet despite these concerns, the cohort has survived for almost 30 years.

**Methods:**

In this retrospective qualitative case study, we examine the community engagement practices of the MOCS and explore the factors that account for its durability.

**Results:**

Women in sex work in Kenya were a highly stigmatized and disfranchised community. As a result, there was no natural 'community' of sex workers either in Nairobi or in the Majengo village. The Majengo clinic aimed to reduce the barriers to health care the women experienced at the STC clinic by bringing the services closer to them and by providing a non-discriminatory environment. The women acknowledged the fact they had hoped their participation in the MOCS would have helped them find a path out of the sex trade. But our findings also add another dimension to this debate, since every cohort member we interviewed expressed her gratitude for the deep impact the MOCS has had on her life, much of it beyond the improved health status made possible by access to quality healthcare services. Participation in the MOCS has improved and enriched their lives. The CE activities have played a central role in creating a community that did not exist independently of the MOCS.

**Conclusions:**

Our case study identified 3 distinct phases of community engagement in the MOCS: (1) reaching out: mobilization, dialogue and education; (2) foundations of trust through relationships of care; and (3) leveraging existing social capital to form a cohort community. The findings demonstrate the importance of some of the less obvious benefits of participation in research, namely the evolving experience of community and the accompanying gains in personal security and solidarity that have kept the women in the cohort, some for 20 years or more.

## Background

There is broad agreement within the biomedical research and bioethics communities that community engagement (CE) is an ethically significant aspect of research [1-3] and experiences with community engagement in research are being reported more frequently in the scientific literature [4-8]. Community engagement has been defined as "the process of working collaboratively with and through groups of people affiliated by geographic proximity, special interest, or similar situations to address issues affecting the well-being of those people"[9]. Our own research program is currently focused on what makes CE effective [10]. To address this central question, we are currently conducting 10 case studies in a wide range of research contexts as part of the Ethical Social and Cultural (ESC) Program for the Bill & Melinda Gates Foundation's Grand Challenges in Global Health Initiative [11].

In this paper, we report the findings of a case study of the CE activities of the Majengo Observational Cohort Study (MOCS), Nairobi, Kenya (see Table 1) [12]. Recently, the MOCS has attracted the attention of journalists,[13,14] bioethicists [15] and HIV/AIDS activists who have emphasized the vulnerability of the sex workers in the cohort, and the fact that participation in the cohort has not provided the women with a path out of the sex trade. Yet, despite these profound challenges, this cohort has been sustained and, indeed, has grown over the past 25 years. This prompted us to study the CE strategies employed in the development of the cohort study to understand the key factors that might explain its durability, in light of some of the recent criticisms.

**Table 1:** A brief overview of the Majengo Observational Cohort Study (MOCS)

•	The MOCS is one of the longest-standing observational cohort studies in the world.
•	It was established in 1984 as a collaborative project between the University of Manitoba, Canada and the University of Nairobi, Kenya with a two-part mandate: (a) to conduct epidemiological research on STIs, and (b) to provide healthcare services to those women diagnosed with active STIs, or their clinical consequences. For more detailed description of the cohort please refer to the work by Kimani and collaborators.^12^
•	It was not originally intended to be a long standing research enterprise.
•	Over time, the MOCS has become renowned for its contributions to the understanding of HIV/AIDS immunology
•	The cohort currently consists of approximately 3,000 women sex workers.
•	Once recruited to the cohort, the women are eligible for a package of free comprehensive health care services.
•	Today, the MOCS contributes at least US$ 1,088/per woman/per year towards their health care.
•	The incidental discovery, in the late 1980s, of HIV resistance among a small number (about 5%) of cohort members, despite their chronic exposure to HIV through unprotected sex, was a turning point for the MOCS and for HIV/AIDS science.
•	The scientific contributions of the MOCS since its inception have attracted numerous academic institutions from the North to join the original collaboration, which has grown into a multi-institutional and multidisciplinary scientific endeavor.
•	The MOCS has produced approximately 300 research papers over the past 25 years, many co-authored by Kenyan and Northern collaborators.
•	To improve the research capacity in Nairobi, the investigators secured a grant from the Canadian Foundation for Innovation (CFI) for CAN $105 M to help build and equip a Level III bio-containment unit along with expansion of existing infrastructure and capacity related to general bacteriology, virology, serology, and PCR. The lab is awaiting commission in January 2010 and will provide an important resource for the region.
•	The Project office in Nairobi is situated at the University of Nairobi which houses research, administration and other support staff; and also computer and life sciences laboratories.
•	The Majengo clinic (MC) serves as the field site of the MOCS in the Majengo village, an administrative unit of the Nairobi City Council in south-east Nairobi.

We describe three phases of evolution of the MOCS CE model, discuss its significance for the MOCS, and conclude with a discussion of the implications of our findings for CE in prospective cohort studies and for CE in global health research more generally.

## Methods

We made an exploratory visit to the MOCS in October 2007 to determine the scope of our case study (Table 2), followed by an eight week data collection visit.

**Table 2:** Preliminary insights and observations

	**Community engagement at the Majengo cohort**
•	The primary goals of CE in the MOCS are to educate sex workers about HIV and other sexually transmitted infections and encourage them to visit the Majengo clinic for healthcare services; to recruit the women to join the cohort; and to retain them in the cohort.
•	In the process, the MC staff engages with the two key constituencies - the existing cohort members, and sex workers in the Majengo village who have not yet joined the cohort.
•	CE activities primarily take place on the MC premises or in the surrounding Majengo village.
•	The CE strategy has grown and evolved over time.
•	There has never been dedicated funding for CE for the MOCS due to common restrictions in research grant budgets.
	**The Majengo clinic (MC): The hub of community engagement**
•	The MC staff normally consists of two physicians, four nurses and one assistant. The physicians perform the clinical screening tasks related to recruitment to the cohort and the nurses and assistant are responsible for the day-to-day running of the MC.
•	The MC serves an important social role as the main space for interaction between the MOCS and the women of the cohort.
•	The MC is the sole experience of the MOCS for most cohort members and other women sex workers who may be considering joining the cohort.

We used a retrospective qualitative case study approach [16] to examine the history of the Project's CE practices over the past 25 years. We conducted 48 in-depth interviews: cohort members including peer leaders (n = 28), MC staff (n = 6), researchers and project leaders (n = 6), research students (3), laboratory staff (2), research ethics board members (2), women's group representatives and others (4). This was complemented by non-participant observation of 4 project *barazas *("meetings" in Swahili). We also examined key documents, such as the informed consent materials provided to prospective cohort recruits at the Majengo clinic, and the standard protocol for health care services to the cohort members.

Prospective interviewees were identified initially by the researchers on the Project and the Majengo clinic staff, and then by sequential referral sampling. They were first approached to briefly introduce the case study and its goals and to ask if they were willing to meet with one of us (SB) for further information about the case study, and potentially to be interviewed.

The interview guide and the consent forms were translated into Kiswahili, the predominant local language spoken by the women of the cohort. All the interviews with cohort members were conducted in Kiswahili, with simultaneous translation provided by a Kenyan translator from Nairobi with experience in social sciences research and gender studies. All the interviews with Cohort members were conducted at the Majengo Clinic, whereas most others were conducted at the project office at the University of Nairobi. Interviews lasted between thirty and 60 minutes. One of us (SB) conducted all the interviews and, accompanied by the translator, made non-participant observations of CE activities, including peer leader meetings and *barazas *of cohort members. Interviews were recorded, translated and transcribed verbatim.

Preliminary data analysis was conducted by SB, with input by JK, during the data collection phase in Nairobi. Initial line-by-line coding of interview transcripts was conducted by SB using the qualitative data software ATLAS.ti version 5.2. The initial coding was followed by focused coding and creation of analytical memos, as described by Charmaz [17]. This process produced a set of thematic and conceptual categories. We developed network views in ATLAS.Ti to explore relationships among the emerging concepts. We discussed and deliberated about these analyses on an ongoing basis. We drafted a preliminary manuscript as a "best fit" for the data and refined it through several subsequent iterations.

The case study research protocol was approved by the respective Research Ethics Boards at the University of Toronto, and the University of Manitoba, Canada; and the University of Nairobi, Kenya.

## Results

### The context: sex work in Majengo

Women are forced into sex work, in Majengo as in the rest of the world, by poverty and adverse social circumstances, including the loss of parents to HIV/AIDS at a young age, domestic violence, and abandonment by partners. These circumstances often result in diminished opportunities for education or access to any vocational training. Women choose sex work because it provides substantially more income than alternative employment opportunities and because they must often provide for their own and extended families.

Sex work remains illegal in Kenya. Women, therefore, must strive to maintain secrecy around their sex work. The women are not organized through brothels or a similar system, as is the case in some other parts of the world. Instead, in the Majengo neighbourhood, they provide their services independently and compete with fellow sex workers to sustain a clientele. Ethnic differences among these women often exacerbate feelings of division and suspicion among the women. These have their origins in the colonial history of the region, when sex workers were imported into Kenya from Tanzania by the British government for its soldiers.

At the time the Majengo clinic was established in the early 80s, activism and advocacy for the rights of sex workers, or any initiative to improve the safety of their work environment, was virtually non-existent. It was a highly stigmatized and disfranchised community with few legal rights or social spaces to organize or appeal for better treatment. This remains true today [18]. As a result, there was no natural 'community' of sex workers either in Nairobi or in the Majengo village. Sex work has long required women in the region to adopt a clandestine approach to many aspects of their lives, working in discrete places, away from their homes and families, and often in cheap rental spaces. This allows them to safeguard their secrets and shield their families from embarrassment and stigmatization. Women's denial of their own identities as 'sex workers' to those outside their clientele continues to present a challenge for effective community engagement.

### The origins of the Majengo Clinic

In the mid 1980s, the Special Treatment Centre (STC) in Nairobi, known as the Casino Clinic by the sex workers in Majengo, because of its proximity to a local casino, was the only public health care centre providing STI care in the city. Through their interactions with the STC patients, a team of researchers from the Universities of Nairobi and Manitoba traced the source of the chancroid epidemic to women engaged in sex work in Majengo village, one of the most impoverished neighborhoods in Nairobi. Faced with the challenge of combating the chancroid epidemic, the public health authority of the Nairobi City Council invited the researchers to help the city address the STI-related public health issues in general, and the chancroid epidemic, in particular. Details about the establishment of the MOCS have been published elsewhere [12,19].

At the time, women engaged in sex work experienced poor and discriminatory treatment at the STC clinic:

...During that time we used to get a lot of gonorrhea ... We would go to town to STC where they would really abuse you and tell you that you were a dog and that is why you keep getting gonorrhea. So we really used to fear going there because you just knew you would be abused. [M32]

... doctors asked us to get our partners [clients]. If we did not get them, they did not treat us. How could we convince our clients to go with us to the Casino clinic? ... [M17]

Because of the poor treatment the patients received at the Casino clinic, the researchers decided to set up a clinic in the heart of Majengo village. A former nurse from the Majengo clinic represented the views expressed by many others we interviewed:

... I think it was Dr. GP1, who came up with the question as to why didn't they go to the place from where these people came and reported the source of infection to women sex workers there? [M39]

The MC aimed to reduce the barriers to health care the women experienced at the STC clinic by bringing the services closer to them and by providing a non-discriminatory environment.

... here [at the Majengo clinic] it is good because they only deal with us sex workers... It is very different. They treat you well. [M34]

In the following sections, we examine the role of CE in the establishment and development of the MOCS.

## Three phases of community engagement at the Majengo cohort study

The MOCS was not originally intended to be a long-standing research project. There was no single period of planning and preparation for CE, but rather a gradual evolution of awareness of the need for specific strategies and practices for CE. We have identified three distinct phases of community engagement as they evolved between 1984 and the mid 1990s: (a) reaching out: mobilization, dialogue and education; (b) foundations of trust through relationships of care; and (c) leveraging existing 'social capital' to form a cohort community.

### Reaching out: Mobilization, dialogue and education

Engaging sex workers in Majengo village required a range of skills and strategies. The cohort study researchers recognized, in particular, the need for an experienced person from the field of community health:

...they [the Project team members] realized that I could mobilize the community, I could get them [women sex workers] together, I could communicate the essence of health seeking behavior that is of benefit to them. And so, they asked me to team up with them to mobilize the women to visit the Majengo clinic. [M09]

Led by the community health expert, the outreach team understood that the women were extremely marginalized, felt disenfranchised, and were often silent about their health concerns, especially STIs. The first formal engagement strategy adopted by the research team, as was described to us by one interviewee, was a 'door-to-door' outreach in the Majengo village and 'one-on-one communication' with the sex workers.

... So it was very challenging to go from door to door trying to figure out how to establish rapport and open a dialogue with a woman. And it is not easy for someone to say that "Yes, I do this [sex work]". But they eventually did come on board. ... [M39]

These initial interactions were also accompanied by a health status benchmark survey to collect data on aspects such as reasons for engaging in sex work and patterns of sex work, women's health status, including STI history and related morbidity, and awareness about safe sex practices. These issues were of obvious relevance and importance to the women and the survey provided an ideal context for the women to reflect on their health and begin a meaningful dialogue with the outreach team. Importantly, the team's community health-oriented approach helped to normalize conversations about STIs and related health risks in the community. Conducting the benchmark survey simultaneously with the outreach work helped enhance the credibility of the MC outreach team since it helped to establish that the outreach team was genuinely interested in the women's health issues and were able to address them in a constructive and non-judgmental manner.

The opportunity was also used to introduce the range of services the MC would provide for the women, including free access to a full range of STI care and treatment, access to free condoms and other forms of contraception, and other related services including health education and one-on-one counseling services. Women continue to have free access to these services even if they decide to leave the cohort.

### Foundations of trust through relationships of care

This first systematic outreach in Majengo village made the research team visible in the community, served as an opportunity for mutual acquaintance with the women, and helped foster a non-judgmental caring attitude for the women by the Majengo clinic staff. The opportunity was also used to introduce the range of services the MC would provide for the women, including free access to a full range of STI care and treatment, access to free condoms, and other related services including health education and one-on-one counseling services. A few women, the "early adopters", visited the MC, were welcomed into the clinic, and their STIs were treated, free of charge. Their initial skepticism began to give way to confidence and trust.

During the initial clinical encounters at the MC, the research aspects of the MOCS were introduced casually to the women, with no formal recruitment strategies or written consent procedures. As one MC staff said:

... we didn't have the consent forms and all that ... the ethics thing was not there! ... it was just verbal, you talk to them ...we explained what would be done at the MC...We used simple language that they (women) would understand, we spoke in Swahili so if they [women] did not understand that they could ask more. ... [M15]

Over time the cohort recruitment procedures have become more elaborate. The major changes include a two-stage process of ascertaining that women work in the sex trade--community based interviewing by peer leaders who are sex workers elected by other members of the cohort to be their representatives followed by additional interviewing by MC staff--a shift from an oral to a written consent, and the development and distribution of standard information materials.

...There are quite a number of activities [that am involved in at the MC]. ...so one is recruitment of the commercial sex workers through the peer leaders. When they come in we give them the information about the clinic, we educate them on HIV and AIDS and STDS. And we provide health education, general health education and then we interview them to ascertain whether they are sex workers. And if we feel that we are comfortable that they are sex workers, then we initiate the process to recruit them into the cohort... [M16]

Many women expressed appreciation for the quality time the MC staff spends while walking them through the process of seeking informed consent and related matters. For example, one cohort member said she liked the informed consent process as '...it is just good to ask for my permission. It is voluntary and so I like it.' [M01].

Almost every woman we interviewed expressed her deep gratitude for being 'cared for' at the MC:

They teach us how to keep our bodies healthy. ...Then they teach us how to live, like about using condoms, how to look after ourselves...the doctors are good and they keep your secrets. If they find you positive (HIV) or negative they keep it secret and that is why I wanted to join... [M27]

...I also like it because I get treatment. I know that if I get any disease or any problem I will be treated. The doctors also make me happy. The way they serve us when you come like the way they welcome you. I also feel free to tell the doctor any health issues I am having. So they just make me happy. [M02]

These experiences of being well cared for stand in sharp contrast to the early reports of discrimination and poor treatment that women experienced prior to the MOCS. It is clear that the women value the access to health care that the MC has made possible for them. But beyond the direct, individual benefits of quality healthcare services, the MC has also offered a much needed 'safe space' for women to share their experiences with one another in a non-judgmental, non-punitive, and respectful environment. This space served as an incubator of new relationships among the cohort members and the MC staff, and provided a social space for non-cohort members to explore this new initiative in their community.

### Leveraging existing 'social capital' to form a cohort 'community'

Social capital refers to a system of networks, norms and trust relationships that enable communities to address common concerns [20 - 22]. It appears that both the MC team and the cohort members--especially early adopters--welcomed the opportunity to build relationships and trust with one another and the MC staff.

As the community began to coalesce around the Majengo Clinic, the early adopters, and other "natural" leaders within the community began to function as a bridge between the clinic and the community:

Some shied [away] a bit but I think now after those who came and got the services I think they sent a word [to the other sex workers] and thereafter they started flowing to the MC ...[M39].

These 'peer leaders' became known for 'giving and sharing with others', 'helping others' and for their 'commitment to the social cause', which earned them the trust of both the women and the clinic staff. These women were intimately familiar with the workings of the community, including the practice of organizing community funds, or "kittys", to provide some financial security in the face of extreme poverty. This "kitty culture" likely provided an important mechanism that contributed to the formation and organization of the research community over time.

... They are able to represent the community better because they live there and they work there. ... [M14]

... Also you see you cannot sell the clinic to other people if you yourself are not getting treatment here. Or you cannot tell someone about the importance of coming to the clinic if you yourself are not coming to that clinic. ... [M21]

Over time, cohort members' and peer leaders' positive word-of-mouth testimonials served as the draw-strings of an extensive social network within the community, pulling women together in a nascent common purpose. This leveraging of social capital began to give form to what would become a long-standing cohort community. Such outreach work, steered by peer leaders and supported by other cohort members, facilitated the gradual accretion of the cohort, which in turn required an increasingly formal approach to the management of outreach work with the Majengo sex workers. Therefore, the role of the peer leaders became more formalized and increasingly integral to the management of the on-going recruitment for the cohort and related outreach work.

Being a peer leader is helping the doctors run the clinic well because of making sure the women come to take part drawing of blood and give other samples that are needed for the research that they are doing. So the clinic would not be there if it were not for the sex workers and the peer leaders help to make sure they attend the clinic. ... So they are important because they make the clinic staff's work easier so they don't have to go out there every day looking for people. Having peer leaders [as part of the system], makes it effective. [M14]

The MC also invested in the peer-leaders more systematically. For example, formal space was created for all peer leaders to periodically meet together with the MC staff to discuss new or controversial issues, prepare for the launch of new satellite projects, and discuss the progress of the research projects. These discussions provided critical insights about the interests of the cohort members and helped the MC develop appropriate communication strategies. These interactions with the MC team strengthened the peer-leaders' legitimacy among the cohort members and provided the peer leaders with a more efficient and authoritative platform for communication with the community.

...Like we have meetings every month as peer leaders and before I come in those meetings, I have to go round to the members and ask them what problems they are experiencing. Then it is me who comes and talks about their problems at the meetings. [M10]

This communication and representation role of the peer leaders was enhanced over time as the initial allocation of space to peer leaders at the MC was expanded to include space for *Barazas *with the cohort community. These meetings, quite literally, brought the emerging cohort community into the MC. The environment and ethos at *barazas *for the cohort members contributed in several other ways to address deeper social issues associated with sex work and its organization. *Barazas *served as an ideal space for the cohort members to learn from one another how to cope with challenges in their work, discuss the potential implications of sex workers' competition for clients, and, for those women who became infected with HIV, an opportunity to learn about the importance of adherence to their prescribed treatment regimens, by meeting women who were HIV positive and living healthy lives on anti-retroviral therapy.

....Actually *barazas *is like a support group. ... So I look at barazas as being very important because ...it [sex work] is a competitive job so people like being selfish, being alone. But these barazas bring us all together. Maybe *barazas *make it possible to have one voice. You even come to learn so many things that you didn't know. Actually, I believe you work as a team. [M29]

These experiences promoted a sense of solidarity and belonging among the women, and provided comfort and security, especially for new entrants into the sex trade. For example, it was this expanded space that helped unite the women on a 'no-condom, no sex services' campaign that has likely averted thousands of HIV infections over the years.

The expansion of available space in the form of *Barazas *at the MC also offered women better opportunities to register their dissenting voices collectively, or individually, with MC staff if they chose to do so.

...they tell me (peer leader) these complaints. I tell them that when they come to the meetings they should complain about it. Also there are the members who are on drugs (ARVs). They would like them [the clinic] to give them foods also like flour and such. Also they say that giving them 200 shillings after they have removed blood, they say [it] is too little. They have said that it should be added to be at least 500 shillings. So they do complain...[M28]

Initially, group leaders were identified by the outreach team, hand-picked by the MC staff, and entrusted with the expanded role that came to be known as the 'peer leader'. Over time, a more elaborate and complex system has evolved whereby the cohort members elect their own peer leaders through a process of candidate 'nominations' followed by 'elections'. These processes required social mobilization within the cohort community and appealed to deeply held values about fair representation, which gradually have shifted the locus of accountability and trust for the cohort members' involvement in the cohort study beyond the MC team to rest with the cohort members themselves.

...there was a peer leader who was there before me and she did not last for very long, it was just for 4 or 5 months. Her behavior was not good, she was a liar and so not trustworthy. She was also a thief and the members did not want her in that position. So in the village we decided that we did not want her so when we came for the meeting we said that we did not want her to be a peer leader any more. So in the next meeting the staff said that we needed to elect somebody else. ... [M14]

Today, after almost 25 years since the establishment of the MC, the outreach work is being managed largely by the peer leaders and supported by the MC staff. There are monthly meetings between peer leaders and the MC staff and collective deliberation and decision making about issues that affect the interests of both parties. The *Barazas*, small, quarterly, and large annual ones for all the cohort members to meet, are the main vehicle for engagement with cohort members, providing opportunities for continuing health education, introducing new projects and dissemination of key research findings, the election of peer-leaders, and to provide space to voice critical opinions and dissent.

## Satisfaction despite unfulfilled aspirations

The women of the cohort expressed disappointment that their participation in the cohort had not resulted in their liberation from the sex trade. The length and depth of the relationship between the women of the cohort and the fact that the research project team has provided the women with so many important benefits, including more formalized representation and generally improved social circumstances, has fostered a sense among the women that the researchers should play a further role in helping them leave the sex trade.

There is something they [cohort members] can do. Because maybe they would like to start a business and stop this work. So I think they [project team] can give us a loan to do this... [M17]

The researchers have made efforts, during the evolution of the cohort, to help the women leave the sex trade. But these have not been successful. Many cohort members offered us their views about why they thought these efforts did not succeed, including inadequate business skills development and related support, and lack of close supervision and accountability for grants provided to women to help them leave sex work.

I think those women can be helped with money but also they must have something to do with that money. They must be supported in businesses that they want, what their heart wants and not what you think that they should do.[M02]

Fundamentally, the problem appears to have three main dimensions: (1) the lack of financial, material and human resources necessary to implement and sustain meaningful skills training and management of career transitions for the women; (2) lack of clarity with respect to the precise obligations of the researchers and their lack of access to dedicated funds and resources to devote to these challenges; and (3) the generally poor state of economic development, and therefore very limited economic opportunities, in the region.

Researchers and MC staff shared the women's frustrations, but also reported frustrations of their own with respect to this profound social challenge:

...We are responding to their health needs very well, no problem. But socio-economic needs? Not that well. If we can respond to those a little bit better it would be good... [M09]

...there is also the unmet need of trying and getting them out of sex work and I have been asked that question many a times. I always ask them back if they could tell us someone who can support these women and help them exit sex work ... [M40]

Despite the acknowledgement by the women and the researchers that removal from the sex work is a fundamental problem, there was universal agreement among the women we interviewed that their participation in the cohort has been beneficial for them.

...I would say that it has impacted my life very positively. [M02]

...I am grateful to them for my good health. ... [M28]

Some of the women emphasized the quality and personalized nature of the information and education they received at the MC, in contrast to information that is delivered through the mass media. Many women told us how highly they valued the treatment they received at the MC, compared to other public health care facilities. They were extremely grateful, in particular, that they were accepted as they were, and deemed worthy of attention, care and health care services at MC. Some women also emphasized that this nurturing environment helped them to appreciate their own 'well-being' and to develop of a sense of empowerment.

## Discussion

Recent commentators have focused squarely on the fact that the women in the MOCS have remained in the cohort, sometimes for decades, and argued that the investigators should have ensured their removal from the sex trade in exchange for their participation in the research. The women we interviewed for this case study acknowledged the fact they had hoped their participation in the MOCS would have helped them find a path out of the sex trade. But our findings also add another dimension to this debate, since every cohort member we interviewed expressed her gratitude for the deep impact the MOCS has had on her life, much of it beyond the improved health status made possible by access to quality healthcare services. Participation in the MOCS has improved and enriched their lives. Although the on-going provision of healthcare services has been of enormous, and obvious, benefit to the women, there is an even deeper contribution that has arisen directly from the way the investigators have approached the CE activities for the cohort. Put simply, the CE activities have played a central role in creating a community that did not exist independently of the MOCS.

The earliest community mobilization activities were consciously organized around community health principles that aimed, in essence, to draw women out of their social isolation in ways that would allow them to receive individual level healthcare--which the investigators and their partners have provided to the women continuously over the past 25 years--but also to facilitate greater recognition of their mutual challenges and to encourage the women to care for one another in a more organized fashion. Although the women lived in close proximity to one another, they did not share 'feelings of solidarity' [23] or 'a sense of belonging together' [24] that serve as the "glue" of a community, regardless of shared characteristics traditionally associated with "community", such as, geography, language, culture, or profession. However, the cohort members--particularly the peer leaders--managed to quickly draw from and build upon existing social networks, resources and capacities within the community of women in sex work in the Majengo village to encourage them to join the cohort study. This experience resonates with the values reflected in the concept of 'relational solidarity', described by Baylis et al. in the context of public health ethics, which recognizes interconnections without relying on assumptions about commonality or collective identity. Instead, 'relational solidarity' concerns the "shared interest in survival, safety and security" [25]. Similarly, Brunger and Weijer [26] have argued that the risks and social implications of any given research program can serve as the common bond that effectively turns disparate individuals into a community for the purposes of research.

The MOCS investigators and the staff of the MC recognized the emergence of this new community and facilitated it in several key ways. First, they recognized that non-discriminatory treatment was essential to help the women view themselves, and each other, first and foremost as women--rather than sex workers engaged in clandestine and illegal activities. This contributed not only to cohort members' health, but also gave them a sense of personal security, a sense of being free from physical and psychological abuse. Second, they understood that the day-to-day practice of community requires space and, for the Majengo women, it was critically important that the space be safe and reliable and that they could have the latitude to make it their own. And so, the dedication of part of the physical space of the MC, even though small, provided the physical substrate for the emerging community. And third, they recognized that with the maturation of the community over time the women had a growing need for greater ownership and formal recognition of their leadership and representation. The MOCS investigators and MC staff responded by facilitating the development of the role of peer leaders, who have assumed a great deal of responsibility for the cohort on a day to day basis.

A central tension arises in the examination of the CE activities of the MOCS in the recognition that sustaining the cohort has clearly benefited the MOCS researchers' interests and careers. This has been a key focus of recent criticism of the MOCS [13,14]. Although this is undeniably true, it is equally true that the scientific insights generated by the MOCS have contributed significantly to the fight against HIV/AIDS, and that the durability and longevity of the cohort itself has been driven by a deep understanding of, and constant attention to, the needs and interests of the Majengo women, while falling short of facilitating their liberation from the sex trade. They are two sides of the same coin. In previous work, we have used the MOCS as a case example to examine the obligations of observational researchers to participants in long-standing cohort studies [19]. More generally, the implications of 'role expansion' for researchers, the increasing demands for them to perform tasks for which they do not have the necessary training, have been recognized, but not fully addressed [27].

Although removing women from the sex trade has been presented by some commentators in stark terms as a clear obligation on the part of the investigators that they have failed to fulfill, our findings suggest a more complex and ethically nuanced situation. Women want to leave sex work, and would like help to do it. And researchers would like to help women, and have made some attempts to do so such as introducing microfinance initiatives and business development training, but these efforts have largely failed because of difficulty securing sustainable funding for these activities and because the grim economic circumstances in the region make alternative avenues of income generation unpopular with the sex workers, even those who would prefer to leave [19]. But overall, there appears to be equilibrium between the recognized need to do more to get women out of sex work and the recognition that the researchers have benefitted the women in real and important ways that extend beyond the scope of their science.

## Limitations

The study has two main limitations. First, the sequential referral sampling method is likely to create a skewed sample of women who's views were well known because they were more vocal and/or articulate than some of the other women in the cohort. As a result, there may have been other perspectives, and in particular, more negative perspectives, that are not as well represented in this report. Second, although we knew early on in the study that very few women have successfully left the cohort, or sex work, during the past 25 years, our sampling strategy did not identify any of these women. These perspectives might have offered some useful critical counterpoint to the uniformly positive views of the participants expressed about their experiences in the cohort, despite their desire to leave sex work.

## Conclusions

Our case study identified 3 distinct phases of community engagement in the MOCS: (1) reaching out: mobilization, dialogue and education; (2) foundations of trust through relationships of care; and (3) leveraging existing social capital to form a cohort community. These phases mark the stages of evolution of a cohort community, drawn together initially by the availability of services and held together over time by 'relational solidarity' and the moral, practical and material support of the researchers. The women of the cohort express strong satisfaction with the benefits they have received over the years through their participation in the cohort, despite the fact that their participation in the research did not result in their removal from the sex trade, as they had hoped.

The findings provide insights into the deep social impact of a long-term prospective cohort study in a resource-poor setting and illustrate the complex balance for investigators between developing and maintaining a cohort for the purposes of research, while providing appropriate care for the women, without dedicated resources. The findings demonstrate the importance of some of the less obvious benefits of participation in research, namely the evolving experience of community and the accompanying gains in personal security and solidarity that have kept the women in the cohort, some for 20 years or more. These benefits provide a new dimension of analysis in the on-going debate about exploitation in research.

## Competing interests

The authors declare that they have no completing interests.

## Authors' contributions

SVSB and JVL originated the study as part of the larger international community engagement research initiative of the Ethical, Social, Cultural Program for the Grand Challenges in Global Health initiative. SVSB was involved in all aspects of its implementation - data collection, preliminary data analysis during the data collection phase, detailed analysis, developing the draft manuscript as a "best fit" for the data, and revising and editing the manuscript. JVL supervised all aspects of its implementation, participated in the detailed analysis, drafting and editing of the final manuscript. SVSB interacted closely with JK during the data collection phase and later towards the end of analysis, for analytic insights and validation of interpretations of the data in the manuscript. All authors helped to conceptualize ideas, interpret findings, and review drafts of the article. All authors read and approved the final manuscript.

## Pre-publication history

The pre-publication history for this paper can be accessed here:

http://www.biomedcentral.com/1471-2458/10/630/prepub
